# A Fusion Deep Learning Model for Predicting Adverse Drug Reactions Based on Multiple Drug Characteristics

**DOI:** 10.3390/life15030436

**Published:** 2025-03-10

**Authors:** Qing Ou, Xikun Jiang, Zhetong Guo, Jiayi Jiang, Zhanpeng Gan, Fangfang Han, Yongming Cai

**Affiliations:** 1 School of Medical Information and Engineering, Guangdong Pharmaceutical University, Guangzhou 510006, China; oq1007918@163.com (Q.O.); jiang_xikun99@163.com (X.J.); gztguitar@163.com (Z.G.); jiangjiayixinxiang@163.com (J.J.); 2122241020@gdpu.edu.cn (Z.G.); 2 NMPA Key Laboratory for Technology Research and Evaluation of Pharmacovigilance, Guangzhou 510300, China; 3 Guangdong Provincial Traditional Chinese Medicine Precision Medicine Big Data Engineering Technology Research Center, Guangzhou 510006, China

**Keywords:** ADRs, multiple drug characteristics, transformer, graph attention network, graph convolutional network, drug–drug similarity

## Abstract

Artificial intelligence (AI)-assisted prediction of adverse drug reactions (ADRs) has significant potential for improving drug safety and reducing financial costs. Early studies often relied on limited dimensions such as the molecular structure of drugs or interactions with biomolecules. In contrast, integrating these characteristics provides valuable insights into ADR predictions from multiple perspectives, enhancing the comprehensiveness and accuracy of the prediction models. In addition, previous studies have focused on whether a specific adverse drug reaction occurs with a particular drug, ignoring the fact that multiple adverse drug reactions may occur concurrently with a single drug. To address these, we developed a predictor that identifies ADRs early in drug discovery, using a deep learning model designed to fuse multiple drug characteristics. Our approach employed four modules to extract one- and two-dimensional sequence structure information of drug molecules, drug–protein interaction data, and drug similarity. A fusion model integrated these characteristics to predict the precise probability of ADRs. The receiver operating characteristic–area under curve (ROC-AUC), area under precision–recall curve (AUPR), and F1 scores on the benchmark dataset are 0.7002, 0.6619, and 0.6330, respectively. The AUPR is significantly improved compared to the conventional multi-label classifier (from 64.02% to 66.19%). In addition, we compared the results with the state-of-the-art methods on LIU’s dataset and the AUPR increased from 34.65% to 68.82%, which shows that our model outperforms them in terms of accuracy and robustness. Ablation experiments further validated the effectiveness of the individual modules. This model accurately predicted the probability of various ADR classes by integrating comprehensive information, thereby offering significant value in enhancing monitoring measures for new drug development and clinical use.

## 1. Introduction

Adverse drug reactions (ADRs) are harmful responses to medications administered at standard doses, making their prediction critical for drug development. ADRs pose a significant threat to both individual and public health worldwide. They are a leading cause of death [1], with nearly 800,000 patients globally affected each year, accounting for 3.6% of all hospital admissions [2]. Additionally, ADRs exacerbate medical errors, imposing substantial clinical and economic burdens on healthcare systems and society [3]. Timely and accurate detection of ADRs is crucial for preventing these reactions and reducing healthcare costs [4]. Predicting ADRs not only enhances patient safety but also offers valuable insights for drug development, enabling researchers and pharmaceutical companies to effectively mitigate potential risks and tragedies.

Identifying all potential ADRs during pre-marketing clinical trials is challenging because of time and cost constraints, necessitating post-marketing efforts to detect ADRs through various approaches [5,6,7,8]. In recent years, deep learning models have demonstrated significant potential in capturing complex drugs and their side effect characteristics [9,10,11] due to the influence of the chemical nature of drugs and human body differences, resulting in the occurrence of adverse drug reactions in a variety of manifestations and a wide range of forms, and as deep learning can deal with massive amounts of data, the use of multi-layer neural networks to automatically learn and extract drug features for the prediction of adverse drug reactions has been explored. Many researchers have achieved good results in the prediction of adverse drug reactions through deep learning methods. For instance, Dey et al. [12] converted drug chemical fingerprints into 2D or 3D graphical structures, compressed them into feature vectors using convolution, and applied fully connected neural networks to predict drug side effect associations. Mantripragada et al. [13] utilized SMILES to extract the chemical structure characteristics in a matrix format for input into a Drug Convolutional Neural Network (DCNN) to predict ADRs. Similarly, Lin et al. [14] proposed a model called MoLFormer-XL for encoding the molecular features of typical SMILES, which was then combined with a convolutional neural network (CNN)-based model for predicting adverse drug reactions, and the model outperformed conventional models in previous studies.

Drug–target interactions are critical for predicting side effects [15,16,17]. Although drugs can achieve pharmacological effects by binding to biological targets such as enzymes, receptors, or other protein molecules, these interactions may trigger adverse reactions. For instance, certain drugs may cause side effects by altering metabolic pathways or physiological functions through the inhibition or activation of specific enzymes or receptors. Understanding the binding properties of drugs and target proteins helps to elucidate drug toxicity mechanisms, explore the intrinsic causes of diseases, and improve the accuracy of ADR predictions. LaBrute et al. [18] docked small molecule drugs to a virtual panel of protein targets and then trained L1 regularized logistic regression models on the docking scores to predict side effects. However, existing studies have been limited to the utilization of information from a single dimension for ADR prediction. Integrating multiple drug characteristics such as structural properties and target interactions can provide comprehensive information and aid in uncovering the underlying causes of ADRs. However, such multi-characteristic systems require ingestion, interpretation, and inference of data from diverse sources. Multi-characteristic drug approaches present challenges in terms of data representation, modal transformation, relationship measurement between different characteristics, integration of characteristic information, and knowledge transfer to predictive models. Developing a fusion deep learning method tailored for ADR prediction is anticipated to enhance accuracy and expand the applicability of computational methods, thereby offering significant practical value.

Side effect predictions can be inherently a multi-label learning task, as drugs often cause multiple adverse effects simultaneously [19]. However, this area has received little attention. Traditional prediction methods primarily rely on binary classification models [20,21,22,23], which can predict whether a specific drug will cause a particular side effect. However, these methods ignore the correlation between drugs and multiple adverse reactions, limiting their ability to capture the diversity and complexity of drug side effects. To address this issue, we proposed a probabilistic prediction framework based on multi-label classification, treating ADRs as a multi-label learning task. This approach enabled the identification of all potential adverse reactions associated with a drug and predicted the probability of each reaction, offering more accurate and comprehensive side effect predictions.

This study developed an innovative fusion model to enhance ADR prediction. This model integrated data from multiple drug characteristics to fully exploit multi-dimensional drug-related information. It employed four feature extraction branches: transformer [24] and a Graph Attention Network–Graph Convolutional Network Combination (GAT-GCN) [25] to analyze drug properties, GCN [26] to extract drug–protein interactions, and Tanimoto coefficients [27] to compute drug–drug similarity. These were efficiently integrated to generate precise probability predictions for ADRs across various drug classes using a multi-label probability predictor. The model not only predicted ADRs for individual drugs but also calculated the probability of each potential side effect, improving the accuracy and precision of ADR prediction and offering a more comprehensive and reliable assessment of drug safety.

## 2. Materials and Methods

### 2.1. Datasets

This section first provides details on the SMILES string data, ADRs data, and drug–protein interaction data, followed by a discussion on the integration of these data for training and testing the proposed method.

SMILES string data: These provided a one-dimensional representation of a drug molecule using predefined substructures. The SMILES string for each drug describes its atomic, bonding and molecular structure in detail. A total of 1430 drug samples were extracted from the PubChem [28] database. The Pubchem source can be found at https://pubchem.ncbi.nlm.nih.gov/ (accessed on 10 February 2024), where drugs cover several classes, including antibiotics, antivirals, anticancer drugs, etc., which have a significant impact on the mechanism of action and adverse effects of drugs.

ADRs data: Data related to ADRs were extracted from the SIDER [29] and ADReCS [30] databases. The SIDER database is available at http://sideeffects.embl.de/download/ (accessed on 10 February 2024); the ADReCS data source is available at https://bioinf.xmu.edu.cn/ADReCS/download.jsp (accessed on 10 February 2024). The types of ADRs in both databases cover a wide range of ADRs from mild allergic reactions to severe multi-organ injuries. The types of drugs in the dataset include various types of antibiotics, anticancer drugs, cardiovascular drugs, and neurological drugs, among others. To ensure data quality, we applied the following screening criteria: (1) exclusion of drugs without PubChem IDs, as these are required to obtain drug SMILES from the PubChem database; (2) removal of drugs without SMILES; (3) in order to prevent overfitting of the model and to improve the generalization ability of the model, we censored data with ADRs occurring less than 350 times. Finally, we combined the two data types, which included a total of 1004 drugs, 119 ADRs and 56,477 drug–adverse reaction pairs. The highest labels (ADRs) appeared 889 times, while the lowest labels appeared 353 times; the average number of labels per sample was 56.25, the percentage of unlabeled samples (sparsity) was 0.00%, the percentage of the first 3 high-frequency labels was 4.50%, and the total number of occurrences of the last 10 low-frequency labels was 3589, with a maximum imbalance ratio of 2.5:1.

Drug–protein interaction data: These data were mainly obtained from the BindingDB [31] and Davis [32] databases. The BindingDB source can be found at https://www.bindingdb.org/rwd/bind/index.jsp (accessed on 10 February 2024); the Davis data source can be found at https://github.com/kexinhuang12345/MolTrans/tree/master/dataset/DAVIS (accessed on 10 February 2024). The protein targets involved in the databases include enzymes, receptors, and transporter proteins, among others. These drug targets cover a wide range of biological processes such as immunomodulation, metabolism, neurotransmitter delivery, etc. We retained proteins associated with both drugs and adverse effects, and the final data included 892 protein sequences and 4354 drug–protein pairs.

### 2.2. Multiple Characterization Inputs for Drugs

**Figure 1:**
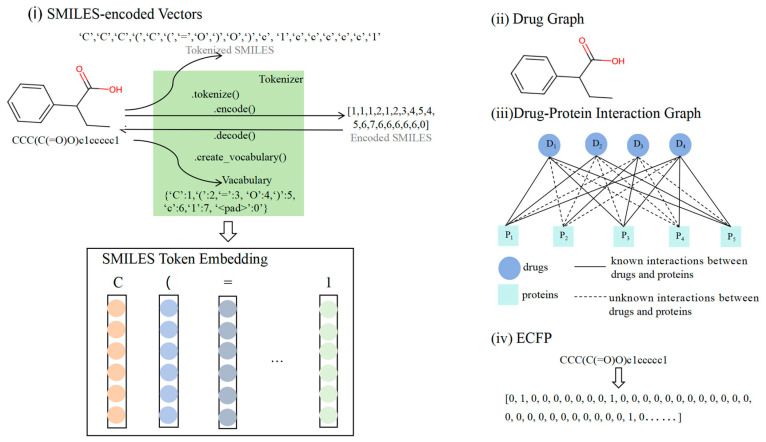
Inputs to our four modules: (**i**) SMILES encoding vector of the drug, (**ii**) molecular diagram of the drug, (**iii**) drug–protein interaction diagram, and (**iv**) Extended-Connectivity Fingerprints (ECFPs) [33] of the drug.

Figure 1 illustrates the multiple characterization inputs for the drugs. Drug structure is a critical representation of chemical properties that can directly affect pharmacological effects and toxic responses. Hence, studying drug structure is essential for ADR prediction. In this study, drug molecules were represented using SMILES-encoded vectors and 2D molecular diagrams. They represent two different but complementary representations of molecules. SMILES encoding, as a linearized molecular representation, is able to compactly encode topological and chemical information about molecules and is suitable for fast input and processing. This representation compactly captures the main information of a molecule’s structure in the form of a string, and is particularly suitable for efficient molecular searches and preliminary screening. However, SMILES encoding lacks a precise description of the three-dimensional structure of the molecule and the spatial relationships between atoms, and thus may have difficulty in adequately capturing complex intramolecular interactions and spatial conformations. In contrast, 2D molecular maps are capable of describing inter-atomic relationships and spatial distribution in detail. By means of graphical structure, 2D molecular diagrams accurately represent the relationships between atoms and chemical bonds in a molecule, and are capable of reflecting more complex structural features, such as ring structures and stereochemical information. This makes 2D molecular diagrams more advantageous in describing interactions within molecules, chemical reactions, and the learning of molecular conformations. We believe that combining these two data representations can provide more comprehensive molecular characterization for models. The advantages of simplicity and fast processing of capturing the molecular structure through SMILES encoding and the deeper structural information provided by using 2D molecular maps can help models understand the multidimensional features of molecules more accurately in drug characterization learning, thus improving the predictive and generalization capabilities of the models. Additionally, drug–protein interaction maps and drug similarity matrices can be included as input features. Drug–protein interactions can elucidate the mechanisms through which drugs may induce side effects via biological pathways, and structural similarity analysis aids in predicting the potential risks of new drugs by comparing the side effect profiles of existing drugs.

#### 2.2.1. SMILES

SMILES (Simplified Molecular Input Line Entry System) is a linear text format for representing chemical molecule structures using simple characters and rules, such as the element symbols for atoms, “=” and “#” for double and triple bonds, the numbers for ring closures, parentheses for branching, and ‘@’, ‘/’, and ‘\’ for chiral and double-bond stereochemistry to describe the atomic connections and arrangements. In the transformer, we set the length of SMILES to 128, and sequences of SMILES of different lengths were truncated or padded to the preset length and mapped to fixed-length integer sequences. This operation was carried out to ensure uniformity of the input data for batch processing and computational efficiency. While we understand the complexity of the chemical structure and that truncation may result in some information loss, by choosing a reasonable maximum length (e.g., based on the molecular size distribution in the dataset), we were able to ensure that the critical information for the majority of molecules was retained without a significant impact on prediction performance. Each symbol in the SMILES sequence can be encoded to generate embedding vectors, and the length of the encoding vector is illustrated in Figure 1i. These embedding vectors were subsequently used as model inputs.

#### 2.2.2. Two-Dimensional Molecular Map

Molecular graphs provide a more natural representation of molecular topologies, and with the rapid development of Graph Neural Networks (GNNs) [34], researchers have been increasingly inspired to use molecular graphs for molecular representation learning. Several graph-based methods have been proposed [35,36,37], demonstrating superior performance over SMILES-based methods for various downstream tasks.

The drug molecules were represented as graphs to provide more accurate molecular structure information [25]. A molecular graph consists of nodes and edges, where nodes represent atoms with various chemical characteristics such as elemental species, electronegativity, valence, and hybridization states. These characteristics are reflected in properties such as the fact that carbon atoms typically form four bonds, hydrogen atoms form one bond, and oxygen atoms form two bonds. The edges represent chemical bonds between atoms, which can vary in type, such as single, double, triple, or aromatic bonds. In addition to connecting atoms, edges can convey other physicochemical properties such as bond strength and length. This structure enables the molecular graph to effectively and precisely represent the structure and properties of a molecule, as shown in Figure 1ii.

The drug–protein graph, shown as (iii) in Figure 1, utilizes a graph representation of both the drug molecule and protein structure. The graph data, including the node characteristics and edge indices, served as the input. Each node represents a drug or protein point and contains corresponding characteristic information, such as atoms or amino acid residues. The edges represent the chemical bonds or spatial proximity between nodes.

#### 2.2.3. ECFPs

ECFPs are topological fingerprints that capture the local structure of a molecule by iteratively identifying the characteristics of atoms and their neighbors, generating a hashed integer identifier for each unique substructure. These identifiers form a fixed-length binary bit vector, where each bit represents the presence or absence of a specific substructure in the molecule (Figure 1iv). ECFPs are widely used in molecular similarity analysis, structure–activity modeling, quantitative structure–activity relationship (QSAR) analysis, and drug screening. The radius parameter controls fingerprint complexity, typically taking values such as 1, 2, or 3, and indicates the different ranges of neighboring structures considered. Software tools for computing ECFPs include RDKit [38], CDK [39], and Chemaxon JChem [40]. In this study, RDKit was used to transform molecules into ECFPs.

### 2.3. Overview of the Framework

**Figure 2:**
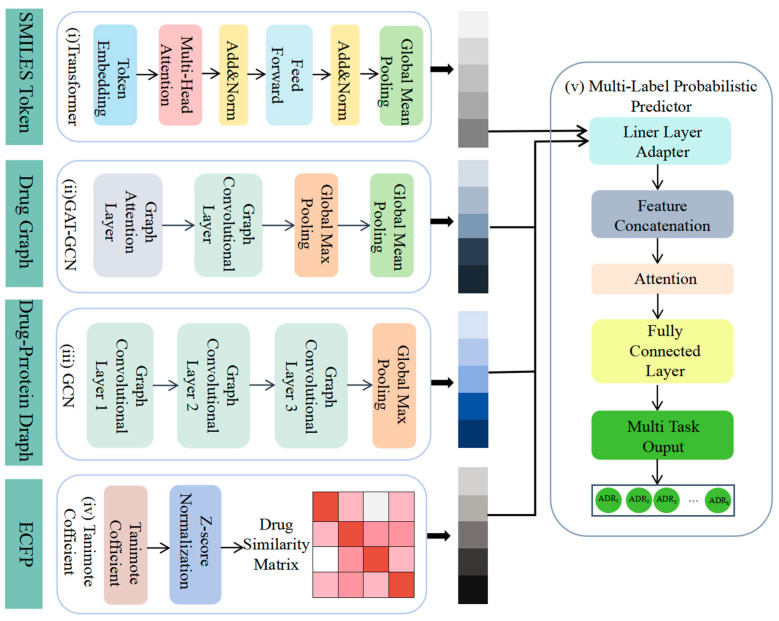
Workflow of our model. We utilized multiple datasets from various open sources: (**i**) a simple transformer encoder to extract drug characteristics, (**ii**) GAT-GCN to learn graph characteristics of drugs, (**iii**) GCN to extract drug–protein interaction characteristics, (**iv**) Tanimoto coefficients to compute drug similarity, and (**v**) integration of four characteristics for multi-label probability prediction of adverse drug reactions by multi-label probabilistic predictors.

We proposed a novel fusion deep learning architecture for the multi-label probabilistic prediction of ADRs (Figure 2). First, SMILES data were utilized as input, and the drug characteristics were extracted using a simple transformer encoder (i). The SMILES representation of the drug was transformed into a molecular graph and its graph characteristics were learned using GAT-GCN (ii). The drug–protein interaction characteristics were then extracted using GCN (iii), and the drug–drug similarity was calculated using Tanimoto coefficients (iv). Finally, the four characteristics were integrated as inputs to the multi-label adverse drug reaction probability prediction model (v), which predicted the probability of adverse reactions for different classes of drugs.

#### 2.3.1. Transformer Is Used to Extract One-Dimensional Sequence Information of Drugs

The transformer model consists of an embedding layer, a transformer encoder layer, and a fully connected layer. For drug representation *x*, after data transformation and position encoding, the characteristics were extracted by the encoder to predict the probability of adverse reaction labels. First, the input integer ID sequence *H* = [*x*_1_, *x*_2_, *x*_3_, …, *x_n_*] is converted into a high-dimensional vector, where each discrete symbol *x_i_* is transformed into a continuous vector representation *E*(*x_i_*) by the embedding layer.

The transformer encoder is the core component of the model and consists of multiple encoder layers. Each layer includes two main sublayers: a multi-head self-attention mechanism and a feed-forward neural network. The multi-head self-attention mechanism enables the model to consider the relationships between each position and the others in the input sequence. By using multiple attention heads, the model can focus on different parts of the input from various perspectives, thereby capturing richer contextual information. The mechanism generates three vectors for each input word, such as the query vector (Q), the key interrogator (*K*), and the value interrogator (*V*), by multiplying the word-embedding vectors (*H*) with three matrices (*W_q_*, *W_k_*, and *W_v_*) that are learned during training. The computational procedure of the multi-head self-attention mechanism is as follows [41]:

(1) $$Q,K,V={HW}_{q},{HW}_{k},{HW}_{v}$$

(2) $$\mathrm{Attention}\left(Q,K,V\right)=\mathrm{softmax}\left(\frac{{QK}^{T}}{\sqrt{d_{k}}}\right)V$$

where *HW_q_*, *HW_k_*, and *HW_v_* are learnable weight matrices, *T* is the transpose of *K*, and *d_k_* are the dimensions of the key.

The multi-head self-attention mechanism can improve self-attention by running multiple independent self-attention mechanisms in parallel and then combining the results. This process is calculated as follows [41]:

(3) $$Q^{\left(h\right)},K^{\left(h\right)},V^{\left(h\right)}={HW}_{q}^{\left(h\right)},{HW}_{k}^{\left(h\right)},{HW}_{v}^{\left(h\right)}$$

(4) $${head}^{\left(h\right)}=Q^{\left(h\right)},K^{\left(h\right)},V^{\left(h\right)}$$

(5) $$\mathrm{MultiHead}\left(H\right)=\left[{head}^{\left(1\right)}\ldots {head}^{\left(n\right)}\right]W_{o}$$

where *n* is the number of heads; the superscript *h* denotes the index of the head; and $\left[{head}^{\left(1\right)}\ldots {head}^{\left(n\right)}\right]$ denotes the last dimension of the join. Typically, $d_{k}\times n=k$ denotes the size of $\left[{head}^{\left(1\right)}\ldots {head}^{\left(n\right)}\right]$ as $R^{l\times d}$; and *W_o_* is the learnable parameter of size $R^{d\times d}$.

The output of the self-attention layer is passed through a feed-forward neural network that performs nonlinear transformation of the input vector. This network typically consists of two linear layers with a ReLU activation function in between, enhancing the model’s expressive power. This process can be represented as follows [41]:

(6) $$\mathrm{FFN}=\max\left(0,{xW}_{1}+b_{1}\right)W_{2}+b_{2}$$

where *W*_1_, *W*_2_, *b*_1_, and *b*_2_ are learnable parameters, *W*_1_ is the weight matrix from the input layer to the hidden layer, *W*_2_ is the weight matrix from the hidden layer to the output layer, *b*_1_ and *b*_2_ are the bias terms, with $W_{1}\in R^{d\times d_{ff}}$, $W_{2}\in R^{d_{ff}\times d}$, $b_{1}\in R^{d_{ff}}$, and $b_{2}\in R^{d}$, and $d_{ff}$ is a hyperparameter that specifies the dimension of the hidden layer in the feedforward neural network, that is, the number of neurons in the hidden layer.

Residual connectivity [42] and layer normalization were incorporated between encoder layers to enhance the training stability and performance of the model. Residual concatenation helps mitigate the vanishing gradient problem, whereas layer normalization addresses the “covariate shift” issue by renormalizing the computed vector representation. Finally, the encoder output is pooled by a pooling layer to obtain a fixed-length vector for the subsequent operations.

#### 2.3.2. GAT-GCN Is Used to Learn Two-Dimensional Structural Information of Drugs

We used the GAT-GCN to learn the graph representation of drugs. GAT-GCN combines GAT [43] and GCN [26] to process graph-structured data, effectively capturing the complex relationships between a drug and a biomolecule. The SMILES strings were converted into graph structures using the RDKit library to extract the atomic and chemical bonding characteristics of drugs as inputs. The model consisted of multiple GAT, GCN, and pooling layers.

GAT introduces an attention mechanism that enables nodes to weigh their neighbors based on their importance when aggregating their characteristics. The core idea is to compute the attention weights for each pair of neighboring nodes and use these weights to adjust the aggregated characteristics. We used three GAT layers to capture the local dependencies between drugs. The GAT layers adopted a set of node features as inputs and applied a weight matrix $W\in R^{F^{\prime }\times F}$ to linearly transform each node, where *F* refers to the dimension of the input features, and *F*’ refers to the dimension of the features after some transformation. A shared attention mechanism $\alpha :R^{F^{\prime }}\times R^{F^{\prime }}\to R$ was then applied to the nodes [43].

(7) $$e_{ij}=\alpha \left(W\vec{h_{i}},W\vec{h_{j}}\right)$$

$e_{ij}$ denotes the importance of the characteristics of node *j* to node *i*, $\vec{h_{i}}$ and $\vec{h_{j}}$ are the feature representations of nodes *i* and *j*. To ensure that the coefficients were easily comparable across different nodes, we normalized them across all choices of *j* using the softmax function [43]:

(8) $$\alpha_{ij}={\mathrm{softmax}}_{j}\left(e_{ij}\right)=\frac{exp\left(e_{ij}\right)}{\sum_{k\in N_{i}}exp\left(e_{ik}\right)}$$

In our experiments, the attention mechanism α was implemented as a single-layer feed-forward neural network, parameterized by weight vectors $\vec{a}\in R^{2F^{\prime }}$, with LeakyReLU nonlinearity applied (negative input slope α = 0.2). The coefficients computed by the attention mechanism are fully expanded as follows [43]:

(9) $$\alpha_{ij}=\frac{exp\left(\mathrm{LeakyReLU}\left({\vec{a}}^{T}\left|W\vec{h_{i}}\right|\left|W\vec{h_{j}}\right|\right)\right)}{\sum_{k\in N_{i}}exp\left({\vec{a}}^{T}\left|W\vec{h_{i}}\right|\left|W\vec{h_{k}}\right|\right)}$$

where ·T denotes the transpose, and ‖ denotes the join operation.

After obtaining the normalized attention coefficients, a linear combination of the corresponding characteristics was computed as the final output characteristics of each node with the potential application of the nonlinear coefficients *σ*. To stabilize the learning process of self-attention, a multi-head attention mechanism was employed to enhance the model’s expressive power [43]:

(10) $$\vec{h_{i}^{\prime }}={∥}_{k=1}^{K}\sigma \left(\frac{1}{K}\sum_{k=1}^{K}\sum_{j\in N_{i}}\alpha_{ij}^{k}W^{k}\vec{h_{j}}\right)$$

where || represents concatenation, $\alpha_{ij}^{k}$ is the normalized attention coefficient computed using the *k*th attention mechanism ($\alpha^{k}$), and $W^{k}$ is the weight matrix of the corresponding input linear transformation.

After the GAT layer, two GCN layers were applied to further extract node characteristics. The core idea of GCN was to update the feature representation based on its neighboring nodes. The update rules for the GCN are as follows [26]:

(11) $$H^{\left(l+1\right)}=\sigma \left({\tilde{D}}^{-\frac{1}{2}}\tilde{A}{\tilde{D}}^{-\frac{1}{2}}H^{\left(l\right)}W^{\left(l\right)}\right)$$

where $\tilde{A}=A+I_{N}$ is the adjacency matrix of the added self-connected undirected graph *G*; *I_N_* is the unit matrix; ${\tilde{D}}_{ii}=\sum_{j}{\tilde{A}}_{ij}$ and $W^{\left(l\right)}$ is the inter-layer trainable weight matrix; σ(·) denotes the activation function, e.g., ReLU(·)=max(0,·); and $H^{\left(l\right)}\in R^{N\times D}$ is the layer *l*th activation matrix; and $H^{\left(0\right)}=X$.

To aggregate the graph characteristics into a fixed-length vector, we applied Global Max Pooling (GAP) and Global Mean Pooling (GMP) after the final GCN layer, resulting in a fixed-length output vector.

#### 2.3.3. GCN Used to Extract Drug–Protein Interaction Characteristics

Although numerous studies have explored the general association between drugs and their side effects, they often fail to investigate the specific mechanisms through which drugs induce side effects, particularly at the level of drug targets and biological pathways [44,45,46,47,48]. The study of drug–protein interactions, as a crucial aspect of understanding drug action mechanisms, helps clarify the causal relationship between drugs and their side effects. By systematically analyzing drug–target protein interactions, we explored how drugs could mediate the biological effects of specific proteins and provided a more precise biological foundation for predicting adverse drug effects.

In this section, we first defined a build graph function that uses the Morgan fingerprint of the drug and the characteristic vector of the protein to construct graph data containing node characteristics and edge indices. The input graph data were then processed through three consecutive GCN layers, each activated by a ReLU function, to extract the graph representation characteristics of drug molecules and protein structures. Finally, a global maximum pooling layer was applied to obtain the graph representation vectors.

#### 2.3.4. Tanimoto Coefficient to Calculate Drug–Drug Similarity

Additionally, recognizing that drug similarity can offer valuable information for capturing potential interaction patterns, we used the Tanimoto coefficient to quantify structural similarity between drugs. Specifically, we first converted the SMILES fingerprint of each drug into a fingerprint object in RDKit and then constructed a drug similarity matrix *S* by traversing all drug pairs. The Tanimoto coefficient was calculated as follows [27]:

(12) $$TC\left(A,B\right)=\frac{\left(A\cap B\right)}{\left(A\cup B\right)}$$

where A and B are the fingerprint vectors of two drugs, respectively, $\left(A\cap B\right)$ denotes the number of characteristics appearing in the intersection of fingerprints A and B, and $\left(A\cup B\right)$ denotes the number of characteristics in the union of fingerprints A and B. The TC ranges from 0 to 1, with 0 indicating the “maximal dissimilarity” and 1 indicating the “maximal similarity”. In addition, because there may be some small errors or floats in the calculation process, which will cause some elements in the matrix to be slightly asymmetric, we ensure the symmetry by averaging the upper and lower triangles and Z-score standardization of the whole similarity matrix, which is a process of standardization by subtracting each similarity value from the mean of that row and dividing it by the standard deviation of that row so that the similarity value of each row is mapping to the same scale to ensure the values in the matrix have zero mean and unit variance, which can eliminate the scale difference of similarity values between different drugs, and thus the similarity of different drugs can be compared under the same scale, which means one can finally obtain the standardized similarity matrix *S*.

#### 2.3.5. Multi-Label Probabilistic Predictor for Drug Characteristics and Adverse Reaction Probability Estimation

To fuse the characteristics of different models, we designed a multi-label probabilistic prediction model that integrated feature adaptation, an attention mechanism, and a multi-task output layer to process multiple input characteristics and predict various tasks. Initially, we used an adapter layer to align the drug–protein graph, drug structure, and drug text characteristics extracted by different models to the same dimensions (512 dimensions), as expressed by the following equation [49]:

(13) $$S_{i}^{\prime }=\sum_{i=1}^{N}W_{adapter}x_{i}+b_{adapter}$$

where $S_{i}^{\prime }$ is the adapted feature and *x_i_* is the input feature vector, $W_{adapter}$ and $b_{adapter}$ are the weight matrix and bias vector of the adapter, respectively, and *N* is the dimension of the feature. Subsequently, the input characteristics (*s*_1_, *s*_2_, and *s*_3_) were transformed from 512 to 128 dimensions using a linear layer adapter, and the adapted characteristics were concatenated with a fourth input characteristic—drug–drug similarity (*s*_4_)—to form a high-dimensional feature vector.

In order to make the model more flexible in extracting useful information from different features, we introduce the attention mechanism. We compute the attentional weights of each feature by a sequence of two linear layer transformations, and with the attentional weights of each feature, we perform a weighted summation of each feature to obtain a new feature vector *S*. The weighted feature vector *S* is input to the hidden layer of the deep neural network for further processing. In each hidden layer, the neurons are weighted and summed based on the output of the neurons in the previous layer, combined with weights and biases. Specifically, the output *S_e_* of the hidden layer neurons can be expressed by the following formula [49]:

(14) $$S^{\prime }=\sum_{d=1}^{D}w_{de}h_{d}+b_{E}$$

where *S*^′^ is the weighted sum for neuron *e* of hidden layer *E*, is the output of the current hidden layer neuron *e*; *D* is the number of neurons in the previous layer; $w_{\mathrm{de}}$ is the weight of neuron *d* connecting the neuron d of the previous hidden layer and neuron *d* of hidden layer *E*; $w_{\mathrm{de}}$ is the weight of neuron *d* connecting the neuron d of the previous hidden layer and neuron e of hidden layer *E*; $h_{d}$ is the value of neuron *d* of the previous hidden layer obtained by applying an activation function to the weighted sum for that neuron; and bias $b_{E}$ is the bias of the hidden layer *E*. The output *S*’ will be used as the input to the next layer of neurons until the last layer of the network. In the last layer of the network, we use multiple linear layers to generate the output of the multitasking, providing predictions for adverse reactions.

## 3. Results

### 3.1. Evaluation Metrics

This study evaluated the performance of the classifier using common metrics in classification problems, including F1-score, ROC-AUC, and AUPR, which are defined as follows:

F1-score: the harmonic average of accuracy and recall combines the performance of both metrics, reflecting the model’s overall classification ability.

ROC-AUC: The area under the receiver operating characteristic (ROC) curve, based on the true-positive rate (TPR) and false-positive rate (FPR), measures the model’s performance across different thresholds. The AUC values range from 0 to 1, with higher values indicating better model performance.

AUPR: The area under the precision–recall curve (AUPR) is primarily utilized to evaluate unbalanced datasets. Unlike AUC, which evaluates the overall model performance, AUPR emphasizes the performance of the positive class.

### 3.2. Experimental Setup

In the experiments, the input characteristic dimension for the transformer was set to 512 with eight attention heads, a feed-forward neural network dimension of 2048, and a dropout probability of 0.1. In the GAT-GCN module, the GAT layer adopted multiple attention heads set to 10, with the number of output features from the previous layer matching the input features of the next layer and a dropout probability of 0.2. We performed a multi-label stratified 5-fold cross-validation of known drugs and adverse reactions. The entire dataset was first stratified by category distribution to ensure consistency across folds and then divided into five equal parts. For each iteration, one of the folds was applied as the validation set and the remaining four folds as the training set. This process was repeated five times to ensure that each fold was used as a validation set. The results from all five evaluations were summarized and averaged to evaluate the final performance. Using stratified sampling, this approach ensured that the category distribution in each fold mirrored the overall dataset, reducing bias from imbalanced categories and enhancing the model evaluation stability and reliability. The entire framework was implemented on the PyTorch 2.5.0 platform using GPU hardware.

### 3.3. Model Comparison

#### 3.3.1. Comparison of Model Performance for Different Lengths of Sequences in SMILES

**Figure 3:**
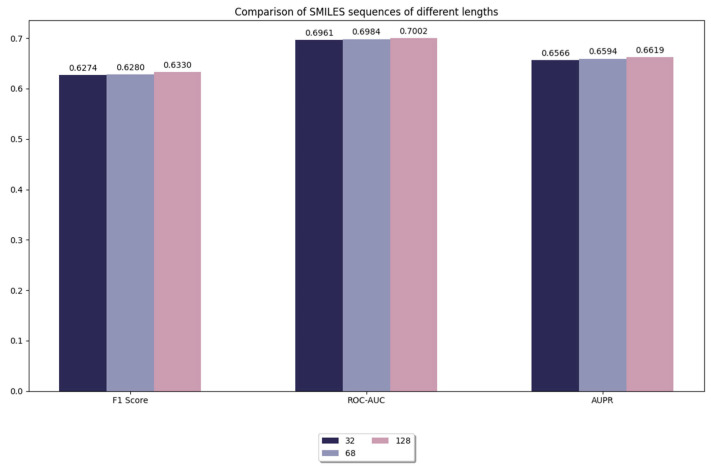
Comparison of model performance for different lengths of sequences in SMILES.

In this experiment, we compared the truncation of SMILES sequences with different lengths, including sequence lengths of 32, 64, and 128. As shown in Figure 3, when the sequence length is 128, the performance of the model is slightly improved compared to shorter sequence lengths (e.g., 64, 32). However, it is worth noting that this improvement is very limited. This suggests that the learning effect of the model is already saturated on our benchmark dataset, and further increasing the sequence length does not result in a significant performance improvement. Although increasing sequence length slightly improves the expressive power of the model, the magnitude of this improvement is already very limited. We believe that the impact of truncation or padding is mainly on the stability and efficiency of model training, while the effect on performance enhancement is relatively small. This may be due to the fact that long sequences are able to retain more chemical information despite the addition of meaningless inputs by padding, which slightly improves the prediction accuracy of the model.

At the same time, we also found that increasing sequence length inevitably brings a significant increase in computational overhead. Longer sequences imply more consumption of computational resources, including more memory requirements and training time. Therefore, when choosing the maximum sequence length, a balance must be found between performance and computational efficiency. Considering the small boost of 128 length and the significantly increased computational burden, we believe that choosing 128 as the maximum sequence length is an effective trade-off. Moreover, 128 is already sufficient to cover most of the chemical information in the dataset. Further increasing the sequence length would provide an almost negligible performance improvement and may lead to a waste of computational resources at the same time. Therefore, choosing 128 as the maximum sequence length not only improves the computational efficiency without significantly sacrificing the prediction performance, but also effectively saves computational resources in practical applications. In summary, 128 as the maximum sequence length achieves a good balance between performance and computational efficiency.

#### 3.3.2. Comparison of Model Performance for Different Data Partition Methods

Data predicting ADRs often exhibit a significant class imbalance, with most patients not experiencing adverse reactions, resulting in fewer samples in the ADR category. In addition, the frequency of different types of ADRs varies widely. To address this issue, we explored various data segmentation methods, including 5-fold, 10-fold, and multi-label stratified 5-fold cross-validations. Table 1 presents the evaluation metric values for these segmentation methods, highlighting the best-performing method for each metric in bold. The AUPR value for multi-label stratified 5-fold was slightly lower than that of stratified sampling 10-fold, possibly because of the larger sample size in each training set, which may reduce the performance of minority class samples. In contrast, 10-fold cross-validation ensured a more balanced proportion of minority class samples in each training set, thereby improving the recall for the minority class. However, the multi-label stratified 5-fold achieved the highest F1-score and ROC-AUC, with improvements of 0.43% and 0.1%, respectively, over the second-best method, the multi-label stratified 10-fold. This suggested that the multi-label stratified 5-fold offered better overall efficiency, stability, and comprehensive classification performance, particularly in the case of class imbalance. The larger training set size in 5-fold cross-validation allowed for more thorough data utilization, reducing the risk of excessive training time and overfitting.

Additionally, the figure illustrates that the performance of K-fold cross-validation was significantly lower than that of the multi-label stratified K-fold. This difference may be attributed to the fact that regular K-fold cross-validation may overlook some class samples in certain folds or result in an imbalanced sample proportion, leading to an inaccurate evaluation. In contrast, the multi-label stratified K-fold ensured that the sample proportion of each category in each fold was consistent with the overall dataset, effectively addressing the issue of class imbalance.

#### 3.3.3. Performance Comparison with Traditional Multi-Label Classification Prediction Models

To validate the effectiveness of the model, it was compared with four traditional multi-label learning models using the Sider and ADReCS databases. The results, presented in Table 2, clearly demonstrate the superior performance of our model across various metrics, significantly outperforming other traditional algorithms in terms of F1-score, ROC-AUC, and AUPR, and highlight its advantages in multi-label classification tasks. Our model achieved an F1-score of 0.6330, which was a 4.29% improvement over the second-highest KNN (0.5901). This improvement was attributed to the adaptive decision regression mechanism, which effectively modeled the label dependencies and optimized the prediction accuracy for each label. In contrast, KNN serves as a nonparametric method, struggling with multi-label problems owing to its inability to capture label correlations by relying on local neighborhood information. For the ROC-AUC, our model scored 0.7002, which was a 3.85% improvement over KNN’s 0.6617. This indicates superior stability in label discrimination across different thresholds. The KNN’s local decision-making strategy, lacking global discriminative ability, resulted in a weaker performance in terms of ROC-AUC. In terms of AUPR, our model outperformed SVM by 2.17%, demonstrating its effectiveness in handling unbalanced categories. The AUPR metric was primarily used to evaluate the model’s performance in handling imbalanced categories, emphasizing the balance between precision and recall. Label imbalance can be a common challenge in multi-label classification tasks, and our model effectively addresses this issue through its adaptive mechanism. By modeling label dependencies and optimizing classification decisions for each label, our model achieved higher precision and recall when processing imbalanced data, outperforming SVM in AUPR metrics. Although SVM excels in certain traditional binary classification tasks, its limited ability to model label dependencies in multi-label scenarios resulted in a comparatively weaker performance in AUPR.

Compared with the decision tree, our model improved by 11.88%, 15.57%, and 16.81% for the three evaluation metrics, respectively. This improvement was likely due to the susceptibility of the decision tree model to noisy data and its limited ability to model correlations between features, which could contribute to its lower performance in the ROC-AUC and AUPR metrics. Decision trees cannot enhance generalization through global optimization, particularly in complex multi-label classification tasks, making their performance significantly inferior. In contrast, Random Forests were 12.5%, 4.62%, and 3.79% lower than our model across the three evaluation metrics. Although Random Forests can improve stability by integrating multiple decision trees, each tree was constructed independently, failing to capture the complex dependencies between labels. Multi-label tasks often involve potential interactions and synergies between labels, which Random Forests struggle to model effectively. In addition, Random Forests are more sensitive to data noise, and complex correlations between characteristics can further affect performance, particularly in high-dimensional data or scenarios with high label correlations. Based on these experimental results, our model significantly improved the recognition of drug reactions compared with traditional multi-label classification prediction models.

#### 3.3.4. Comparison with Existing State-of-the-Art Multi-Label Prediction Models

In this section, we present the evaluation of all methods using Liu’s dataset, which consists of multi-source data with different types of drug characteristics. To evaluate the performance of our model in predicting ADRs, we compared it with five cutting-edge methods: Liu’s method [50], FS-MLKNN [51], LNSM-SMI [52], LNSM-CMI [52], and KG-SIM-PROP [53]. Liu et al. proposed a multi-source approach that incorporated the chemical, biological, and phenotypic features of drugs to construct an SVM classifier for each ADR. However, this method may be limited by feature selection. If the quality or representativeness of certain features is not high, it may lead to unstable performance of the classifier. In addition, building SVM classifiers for each ADR individually may increase the complexity of the model and may be less efficient when dealing with multiple labels or multiple ADRs. The FS-MLKNN identified the key features and constructed a multi-label predictive model consisting of five independent MLKNN sub-models, each trained on a subset of features selected by a genetic algorithm. Selection of feature subsets using genetic algorithms may result in high computational overhead, especially when the feature space is large. In addition, the convergence speed of the genetic algorithm may be slow, which affects the training efficiency. LNSM-SMI and LNSM-CMI are based on the LNSM method, which assumes that the properties of a drug can be reconstructed through linear combinations of its neighboring drugs. The LNSM-SMI method generates K similarity matrices from different K data sources and integrates them in a weighted manner, which may lead to less effective or distorted information fusion, especially if the data sources are of different quality. The LNSM-CMI method, on the other hand, trains the LNSM model on each data source individually, which may ignore the potential correlation between the data sources, leading to insufficient information sharing and affecting the predictive performance of the model, especially when there is a strong interdependence between the data sources. In addition, FS-MLKNN, LNSM-SMI and LNSM-CMI require a large number of neighbors to work properly and at the cost of efficiency due to the generation of neighbors. KG-SIM-PROP constructed a graph structure based on drug similarity matrices and propagated ADR labels from one drug to adjacent drugs. However, this approach may have difficulty dealing with complex, nonlinear relationships, resulting in a model with limited predictive power.

The comparison results (Table 3) indicated that although our model was slightly inferior in terms of ROC-AUC (15.35%, 14.85%, 15.49%, 16.15%, and 16.55% lower, respectively), it outperformed the others in AUPR metrics, surpassing them by 51.16%, 37.73%, 34.17%, 35.50%, and 40.27%, respectively. The ROC-AUC did not fully reflect the performance, particularly when dealing with unbalanced datasets. In ADR prediction tasks, where the negative samples dominated and the ADR labels exhibited a strong imbalance, the ROC-AUC could obscure the ability of the model to identify positive instances (i.e., ADR occurrences). Therefore, although our model’s ROC-AUC performance was slightly lower, this reflected a different strategy to handle the unbalanced dataset, resulting in a significant improvement in AUPR. AUPR more accurately reflects the model performance in imbalanced scenarios, particularly in identifying a few positive instances (ADRs) [54]. Our model’s superior AUPR performance resulted from its integration of multi-dimensional feature information, including one- and two-dimensional drug structures, drug–protein interaction networks, and drug–drug structural similarities, allowing it to comprehensively capture the associations between drugs and ADRs. Although this complexity increased the sophistication of the model, it enhanced its robustness and predictive power, particularly on unbalanced datasets. Consequently, our model avoided the performance degradation caused by an excess of negative samples and demonstrated a clear advantage in the AUPR metric.

In summary, although our model slightly underperformed in relation to the others in terms of ROC-AUC, this difference should not be viewed as a disadvantage. This highlights the strength of the model design. By integrating multi-source features and adaptively optimizing unbalanced datasets, our model enhanced the accurate identification of ADRs, particularly in cases with a severe imbalance between positive and negative samples. The significant improvement in AUPR demonstrated its greater practical value in such scenarios.

### 3.4. Ablation Experiments

Deep Neural Network (DNN) models significantly depend on integrated characteristic information. To validate the superiority of our proposed multimodal attribute strategy for ADR prediction, we thoroughly examined the characteristic information involved. Additionally, to better understand how the model could effectively learn the biological features to predict the occurrence of ADRs and to further validate its performance, we conducted a comprehensive ablation investigation using different settings from our dataset.

1. Remove transformer: we removed the transformer module and used only the molecular structure map embedding learned from GAT-GCN as the drug representation.
2. Remove GAT-GCN: we removed GAT-GCN from the interaction module and used only the one-dimensional substructure sequence information of the drug molecule, extracted from the transformer, as the drug representation.
3. Removal of drug–protein interactions: we further removed the GCN module from the model and extracted only the characteristic information of the drug.
4. Removal of drug–drug similarities: we extracted only the structural information of the drug and drug–protein interaction information.

Table 4 summarizes the results of the ablation experiments, including the F1-score, ROC-AUC, and AUPR values, after removing different modules. These metrics quantified the effect of each module on the overall performance of the model. Specifically, removing the transformer module resulted in F1-score, ROC-AUC, and AUPR values of 0.5624, 0.6147, and 0.5975, respectively, representing decreases of 7.06%, 8.55%, and 6.44%, respectively, compared with the ensemble model. The results indicate that the transformer module is crucial for the performance of the ensemble model. Its removal led to a decrease in classification ability and discriminative power, particularly affecting the F1-score and ROC-AUC metrics. This may be due to the fact that in drug molecules, the relationships between atoms and groups are not limited to local neighborhoods, but may span over more distant atoms and groups, especially in complex molecular structures. The transformer module is able to provide the modeling of such long-range dependencies, which allows the model to understand the interactions of global structural properties of the drug, such as the ring structure and the functional groups. With the removal of the transformer, the model loses the ability to model long-range dependencies, resulting in a model that is not able to effectively capture the global information and complex interactions in the drug structure. As a result, the performance of the model degrades in tasks that require global contextual understanding, and therefore performance is degraded. Similarly, removing the GAT-GCN modules resulted in 6.68%, 8.52%, and 6.38% decreases in the three metrics compared with the ensemble model. This suggested that the combination of GAT and GCN modules was essential for the model, because the main role of the GAT-GCN module is to model the atom-to-atom relationships in drug molecules through graph structures. Molecules are viewed as graph structures, with each node representing an atom and edges representing chemical bonds between atoms. GAT and GCN are able to propagate information through the graph structure, generating a more accurate representation of the molecule by learning the interrelationships between the nodes. By removing this module, the model will not be able to effectively utilize the graph structure information of the drug molecule, losing the depth of modeling the relationships between atoms. The molecular structural properties of the drug will not be accurately represented, which may lead to a significant reduction in the accuracy and generalization ability of the model in tasks such as predicting the biological activity, interactions, etc., of the drug. The F1-score, ROC-AUC, and AUPR for the model without the drug–protein interaction module were 0.5633, 0.6154, and 0.5978, respectively, reflecting decreases of 6.97%, 8.48%, and 6.41%, respectively, compared with the ensemble model. Drugs exert their biological effects by binding to proteins. Modeling of drug–protein interactions is critical to understanding the mechanism of a drug because it can provide clues as to whether a drug can effectively target a specific protein. By learning the interactions between drug molecules and target proteins, the model can speculate on the therapeutic effects, side effects, etc., of the drug. Removing this module means that the model loses its understanding of how the drug acts with the protein. As a result, removing this module significantly decreases the performance of the model, especially when identifying drug-related biomarkers, the model is unable to capture the biological effects of the drug and accurately predict the target proteins of the drug, which leads to a significant decrease in the efficacy of the model. The performance of the model without the drug–drug similarity module further emphasized its importance. Removing this module resulted in decreases of 7.27%, 9.10%, and 6.89% in the three metrics compared with those of the ensemble model. The performance notably declined, particularly in terms of accuracy and robustness, which were lower than those observed when other modules were removed. This indicates that the drug–drug similarity module is essential for drug association modeling. Calculating the similarity between drugs helped reveal their shared characteristics as the model can understand the similarity of certain drugs in terms of their biological effects, which, in turn, can help researchers speculate on the potential effects or side effects of unknown drugs, offering valuable insights for drug combination applications and side effect prediction. These results validated the critical role of drug similarity in enhancing the accuracy and stability of model predictions. Drug similarity plays a crucial role in helping the model capture potential relationships between drugs while effectively reducing noise interference, thereby enhancing the adaptability of the model to new drugs and improving the prediction of complex drug interactions. Without the drug similarity module, the model would not have a comprehensive understanding of the drug population structure and the model would lose the ability to recognize potential relationships between drugs. This means that the model cannot infer the potential effects or side effects of new drugs from the similarity of existing drugs, which reduces the accuracy and interpretability of prediction. Especially in tasks of drug combinations or multiple drug interactions, the lack of drug similarity can seriously affect the performance of the model. After removing this module, the performance of the model is lower than that after removing the other modules, further demonstrating the critical role of drug similarity in improving the accuracy and robustness of the model. Each module in this model had unique and essential functions. The removal of any module significantly reduced the performance, particularly in metrics such as F1-score and ROC-AUC. The results in Table 4 highlight the crucial role of each module in overall performance, demonstrating that the effectiveness of the ensemble model in drug discovery and bioinformatics relies on the synergistic interaction of these modules. The substantial performance loss following the removal of any module demonstrates the integral role of each model component.

Furthermore, we note that removing any of the modules leads to an almost identical decrease in the quality of the final ensemble model, which could be due to several reasons. First, in multimodal learning, models typically synthesize multiple types of input features that are complementary to some degree, and which may provide a similar degree of importance to the prediction task. For example, the chemical structure of a drug, protein interactions, and drug–drug similarities may all contain similar information about drug effectiveness and toxicity. Therefore, removing any set of input parameters can weaken the model’s utilization of this information, resulting in a similar degree of degradation in performance. Second, ensemble models typically consist of multiple submodels, each of which handles a different set of input features. When one of the sets of input features is removed, the model suffers because it may not be able to fully utilize the information provided by those features. However, since the model may have adapted to multiple input modalities, it is still able to make inferences from other features. Therefore, although the removal of one set of features affects the predictive ability of the model, the other features still provide sufficient support, resulting in a more consistent decrease in performance. Finally, in deep learning models, especially when multiple input features are involved, the parts of the model are usually fused with features through gradient optimization during training. When you remove a certain set of inputs, the model may adjust its gradient update strategy to compensate for the missing features. However, this adjustment does not always perfectly complement the missing feature information, resulting in an eventual performance degradation, but the degradation may be similar in magnitude to the removal of other input features. In summary, the input features to the model are complementary, redundant or balanced to some extent. Different input features contribute to the model in a balanced way, and all input features are essential to the final performance of the model, while there is a certain amount of “information overlap” between them, so that the removal of a certain part of the features does not cause an extreme loss of performance but still leads to a decrease in the overall performance.

## 4. Discussion and Conclusions

This study developed a novel deep learning model for predicting the probability of ADRs with multiple labels by fusing complementary drug characteristics using chemical language and molecular maps. The SMILES-encoded vectors were employed to represent molecules, with the transformer encoder and GAT-GCN used for modality-specific learning. To further investigate the mechanism of ADR occurrence, we incorporated drug–protein interactions and drug–drug similarity features. In the process of training and validation, in order to solve the problem of category imbalance and ensure the generalization ability of the model under different data distributions, we adopted stratified sampling 5-fold cross-validation. The model was compared with traditional and state-of-the-art multi-label prediction models, demonstrating superior performance in drug response prediction. Additionally, a series of ablation experiments validated that the proposed model improved prediction accuracy, stability, and noise immunity by utilizing diverse information sources. By extracting and fusing features from the four characteristic extraction models, the model’s ability to handle irrelevant data sources and its generalization ability were significantly enhanced. Multiple-characteristic learning captured the complementarity between different features, which was beneficial for drug discovery. Our model is able to computationally predict adverse drug reactions at an early stage, helping drug developers to assess the potential safety risks of drug candidates at the time of screening and better avoid potential side effects, thus effectively reducing development costs and time. The model enables drug analysis at multiple levels by integrating multiple drug characterization data, including molecular representations, one- and two-dimensional structural information, drug–protein interaction networks, and drug similarity data. It utilizes advanced feature extraction methods, such as network architectures like transformer and GAT-GCN, to deeply mine complex molecular structural information and accurately capture drug–target interactions in organisms. This gives the model a significant advantage in the prediction of adverse drug reactions, especially in the application scenario of multi-drug combinations and their side effect analysis, which can provide accurate prediction results for drug developers. Our study employed multi-label probabilistic prediction to model the adverse reactions of drugs, providing accurate probabilities for ADRs. Compared with traditional dichotomous or single-label prediction, multi-label prediction more accurately reflects the diversity of ADRs, enhances drug safety assessment, offers a more comprehensive risk evaluation. In addition, the combination of drugs (e.g., polypharmacy) is a common therapeutic modality in clinical practice, but there may be drug–drug interactions that lead to the exacerbation of side effects. Our model is capable of evaluating multiple side effects that may be triggered by multi-drug interactions in complex situations. It can provide physicians with a safety assessment of drug combinations, optimize individualized treatment regimens, and reduce the risks associated with drug interactions by predicting drug–drug similarities and drug–protein interactions.

However, the predictive accuracy of the model strongly depends on the quality and completeness of the drug-related data. If the data on the molecular structure of a drug, drug–protein interactions, or drug similarity are incomplete or contain errors, the effectiveness of the model may be significantly affected. Therefore, quality control and accuracy assurance of the data are key factors in the application of the model. Although deep learning models can provide more accurate predictions in drug safety assessment, they are often regarded as “black-box” models, which is challenging in clinical applications. The interpretability of the models remains a key issue. Future work can reveal the mechanisms behind the model predictions by introducing interpretable analysis tools (e.g., SHAP values, LIME, etc.) so that researchers and clinicians can understand how the models arrive at the predictions of adverse drug reactions, thus enhancing their usability and credibility in clinical practice. In addition to these, the current model has some limitations. First, uncertainty remains in predicting drug reactions because drugs may exhibit different behaviors in various individuals or biological environments, complicating accurate predictions. Finally, although the model predicts adverse reactions through multiple labels, its performance may degrade when addressing extreme cases or rare reactions, which requires further optimization. In the future, we can enhance the generalization ability by incorporating additional clinical data dimensions, such as genomic information and environmental factors, and explore the integration of reinforcement learning to optimize the model in real time during various stages of drug development, thereby improving prediction accuracy.

## Figures and Tables

**Table 1:** Comparison of model performance for different methods of data partitioning.

Data Partition Methods	F1-Score	ROC-AUC	AUPR
5-fold cross-validation	0.5486	0.6004	0.5724
10-fold cross-validation	0.5781	0.6381	0.6091
Multi-label stratified 5-fold	**0.6330**	**0.7002**	0.6619
Multi-label stratified 10-fold	0.6298	0.6993	**0.6620**

Bolded numbers are the best performance.

**Table 2:** Performance comparison of different characteristic fusion models.

Models	F1-Score	ROC-AUC	AUPR
Decision Trees	0.5142	0.5445	0.4938
Random Forests	0.5080	0.6540	0.6240
KNN	0.5901	0.6617	0.6064
SVM	0.4392	0.6644	0.6402
Our model	**0.6330**	**0.7002**	**0.6619**

Bolded numbers are the best performance.

**Table 3:** Comparison with existing state-of-the-art multi-label prediction models.

Models	ROC-AUC	AUPR
Liu’s method	0.8772	0.1766
FS-MLKNN	0.8722	0.3109
LNSM-SMI	0.8786	0.3465
LNSM-CMI	0.8852	0.3332
KG-SIM-PROP	0.8892	0.2855
Our model	**0.7237**	**0.6882**

Bolded numbers are the best performance.

**Table 4:** Ablation study of our model under different experimental conditions.

Methods	F1-Score	ROC-AUC	AUPR
Remove transformer	0.5624	0.6147	0.5975
Remove GAT-GCN	0.5662	0.6150	0.5981
Remove drug–protein interaction	0.5633	0.6154	0.5978
Remove drug–drug similarities	0.5603	0.6092	0.5930
Ensemble model	**0.6330**	**0.7002**	**0.6619**

Bolded numbers are the best performance.

## Data Availability

The raw data supporting the conclusions of this article will be made available by the authors on request.
